# Impact of Oral Nutritional Supplementation at Hospital Discharge on Healthcare Costs in Older Adults: A Retrospective Analysis of Japanese Claims Data

**DOI:** 10.1111/ggi.70288

**Published:** 2025-12-26

**Authors:** Masashi Miyawaki, Tatsuya Hosoi, Makoto Yunoki, Seiji Hashimoto, Yoshitaka Kase, Masaki Ishii, Mitsutaka Yakabe, Sumito Ogawa

**Affiliations:** ^1^ Department of Geriatric Medicine, Graduate School of Medicine The University of Tokyo Bunkyo City Tokyo Japan; ^2^ Division of Geroscience, International Center for Brain Science (ICBS) Fujita Health University Toyoake Aichi Japan

**Keywords:** healthcare costs, malnutrition, older adults, oral nutritional supplements

## Abstract

**Aim:**

Malnutrition is a common and costly challenge among hospitalized patients and those requiring postdischarge care. Oral nutritional supplements (ONSs) are a low‐cost intervention for undernourished individuals. This study used the Kokuho Database (KDB) to examine the health economic impact of prescribing ONSs at discharge for hospitalized older adults.

**Methods:**

This retrospective study included patients aged 75 years and older who were discharged from hospitals in Saitama Prefecture, as recorded in the KDB, from April 2017 to March 2022. Patients prescribed ONSs at discharge were classified as the ONS‐at‐discharge (ONSd) group, and those not prescribed were classified as the no‐ONS‐at‐discharge (non‐ONSd) group. Healthcare costs over 360 days postdischarge were compared using propensity score matching.

**Results:**

A total of 526 605 patients were enrolled: 13 042 in the ONSd group and 513 563 in the non‐ONSd group. After matching, 26 084 patients (13 042 per group) were included in the analysis. Over 360 days, healthcare costs were higher in the ONSd group than in the non‐ONSd group (935 092 yen vs. 860 588 yen, adjusted *p* < 0.001). Among patients not prescribed ONS during follow‐up, 360‐day costs remained higher in the ONSd group (882 465 yen vs. 847 700 yen, adjusted *p* < 0.001). Conversely, among patients prescribed ONS during follow‐up, 360‐day costs were lower in the ONSd group (1 040 830 yen vs. 1 185 919 yen, adjusted *p* < 0.001).

**Conclusion:**

Healthcare costs over 360 days were higher in the ONSd group compared with the non‐ONSd group. However, among patients who continued ONS use after discharge, costs were lower in the ONSd group.

## 1 Introduction

Malnutrition, or the risk of malnutrition, is a common and costly challenge, affecting 30%–50% of hospitalized patients and those transitioning home or to another care setting after discharge (postdischarge) [1]. Malnutrition is associated with adverse in‐hospital outcomes, including higher complication rates, prolonged hospital stays, increased hospitalization costs, and increased risks of sarcopenia and functional decline risks [2, 3, 4]. Older adults (≥ 65 years) and patients with chronic conditions, including cardiopulmonary, oncological, or gastroenterological diseases, are at higher risk of malnutrition [5, 6, 7, 8].

Japan's demographic landscape highlights the importance of addressing this issue: adults aged ≥ 65 years constitute 29.3% of the population, far exceeding the average of 18.0% in countries with comparable gross domestic product levels. As malnutrition risk increases with age, improving its identification and management is essential for safeguarding population health and controlling healthcare expenditures.

The nationwide prevalence of strictly diagnosed, disease‐associated malnutrition was 2.9% in Japan [9]; however, malnutrition remains underscreened and underdiagnosed. Malnourished patients incurred two–three times higher monthly healthcare costs than those of comparable members of the general population. The annual economic burden was estimated at 14.5 billion USD, accounting for approximately 4.3% of national healthcare expenditure [9]. Similarly, malnutrition contributes substantially to healthcare expenditure in many countries [10, 11, 12, 13, 14].

Oral nutritional supplements (ONSs) are a simple, low‐cost intervention for patients with malnutrition. ONSs, used either alone or as part of comprehensive nutrition programs, improve the health of hospitalized patients while reducing healthcare costs [15, 16, 17, 18, 19, 20]. Beyond addressing malnutrition during hospitalization, nutritional interventions that include ONSs may help prevent or mitigate age‐related syndromes such as frailty and sarcopenia, which are highly prevalent among older adults [21, 22]. Evidence for frailty primarily comes from multi‐component interventions that include ONSs, whereas in sarcopenia, ONSs have demonstrated benefits even as a standalone approach. Although most existing evidence comes from observational studies, the EFFORT trial—a multicenter randomized controlled study—provided individualized nutritional support during hospitalization, mainly comprising ONSs. The trial demonstrated significant improvements in 30‐day outcomes, including mortality, complications, and healthcare costs, among at‐risk medical inpatients [23, 24].

Hospitalization provides an important opportunity to assess patients' nutritional status and initiate appropriate interventions, in contrast to outpatient care where time and resources may be limited. Based on prior evidence, we hypothesized that prescribing ONS at hospital discharge improves patients' nutritional status and, consequently, is associated with healthcare costs during the postdischarge period.

To test this hypothesis, we conducted a retrospective analysis using a large‐scale claims database of patients aged ≥ 75 years who were capable of oral intake. We utilized the Saitama Prefecture Kokuho Database of Health Insurance Claims and Specific Health Checkups (KDB) to examine ONS use during hospitalization and after discharge and to evaluate its impact on key health outcomes and healthcare costs over a 360‐day postdischarge period. This timeframe allowed assessment of both short‐ and long‐term patterns of ONS utilization and their effects across the care continuum.

Healthcare cost was selected as the primary outcome because it represents a critical, policy‐relevant measure in an aging society and can be comprehensively and reliably captured using claims data. By focusing on this outcome, our study aimed to provide evidence on the economic impact of prescribing ONSs at discharge among older adults.

## 2 Methods

### 2.1 Data Source

This study used data from the KDB, the National Health Insurance database of Saitama Prefecture, Japan. The KDB comprises receipt (claims) data and specific health check‐up records. Receipt data, submitted for reimbursement of insured medical treatments, is compiled and used to support the optimization of medical expenditures and improvement of healthcare quality. Because Japan operates under a universal health insurance system, the KDB includes nearly all older adults covered by the health insurance association of Saitama Prefecture [25]. For this study, we analyzed KDB data from April 2017 to March 2022.

### 2.2 Participants and Follow‐Up

**FIGURE 1:**
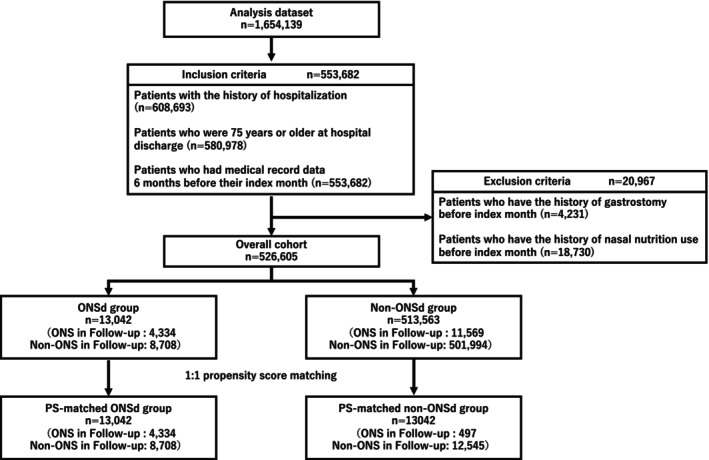
Flowchart for inclusion of participants in the study. ONS, oral nutrition supplement.

Using the Saitama Prefecture KDB, we identified patients who were hospitalized and aged ≥ 75 years at the time of hospital discharge. Patients were included if medical record data were available for at least 180 days before their index hospital discharge date (*n* = 553 682). We excluded those with a history of gastrostomy or nasal nutritional support before the index date (*n* = 20 697). Patients prescribed ONSs at discharge were classified as the ONS‐at‐discharge (ONSd) group (*n* = 13 042), whereas those not prescribed ONSs were classified as the no‐ONS‐at‐discharge (non‐ONSd) group (*n* = 513 563). After propensity score matching, 26 084 patients (13 042 per group) remained eligible for the final analysis (Figure 1).

The date of the first hospital discharge during the study period was designated as the index date. Follow‐up ended at the earliest occurrence of the last available data collection date, 360 days after the index date, the date of first readmission (or the corresponding discharge date for length‐of‐stay analyses), or the date of gastrostomy placement or initiation of nasal nutritional support.

In the KDB, malnutrition was defined using ICD‐10 diagnosis codes. Cases with direct nutritional diagnoses (e.g., E40–E46, E63.9, E77.8, R63.3, R63.4, R64) were classified as strictly diagnosed malnutrition (SDM). Disease categories commonly associated with malnutrition—such as stroke (I60–I69), dementia (F00–F03, G30, G31), chronic obstructive pulmonary disease (J40–J44), depression (F32, F33), and musculoskeletal disorders (M00–M99)—were classified as disease‐associated malnutrition (DAM). For the analyses, SDM and DAM were combined and treated collectively as malnutrition.

### 2.3 Propensity Score Matching

Propensity score matching was conducted to balance characteristics between the ONSd and non‐ONSd groups. Nearest‐neighbor matching was applied at a 1:1 ratio with a 0.2 caliper. Propensity scores were estimated using a logistic regression model that included the following covariates: age; sex; fiscal year of the index date; admission via emergency; comorbidities (malnutrition, stroke, cardiovascular diseases, respiratory diseases, gastrointestinal disorders, psychiatric disorders, endocrine and metabolic diseases, musculoskeletal disorders, hematologic diseases, and immune disorders); total healthcare costs during the 6 months before the index month; drug‐related healthcare costs during the same period; and hospital length of stay (LOS) within the 6 months before the index month. Covariate balance between the matched groups was evaluated using standardized mean differences, with a cutoff value of 0.10. In addition, stabilized inverse probability of treatment weighting (IPTW) based on the same propensity score model was applied as a sensitivity analysis.

### 2.4 Statistical Analysis

Categorical variables were summarized as counts and percentages, and continuous variables were summarized using descriptive statistics (number of patients, mean, standard deviation, minimum, median, and maximum). All statistical analyses were performed using SAS 9.4 (SAS Institute Inc., Cary, NC, USA) and IBM SPSS Statistics 30.0 (IBM Corp., Armonk, NY, USA).

#### 2.4.1 Primary Analysis

Per patient‐healthcare costs—defined as the sum of inpatient, outpatient, and pharmacy claims—were summarized at 30, 60, 90, 180, and 360 days from the index date. To estimate 95% confidence intervals, separate generalized linear models (GLMs) with a gamma distribution and log‐link function were applied for cumulative costs at each time point. To address multiplicity across time points and subgroups, the false discovery rate was controlled at 5% using the Benjamini–Hochberg procedure within each family of tests. Both two‐sided *p* values and Benjamini–Hochberg–adjusted *p* values are reported. As a sensitivity analysis, the same GLMs were fitted to the IPTW‐weighted cohort.

#### 2.4.2 Secondary Analysis

Healthcare utilization per patient—including LOS for readmissions, number of outpatient physician visits, and emergency room visits or emergency calls—was summarized using descriptive statistics over 360 days from the index date. A GLM with a Poisson distribution and log‐link function was used to estimate 95% confidence intervals. Multiplicity was controlled using the same false discovery rate procedure as described in the primary analysis, and both unadjusted and adjusted P values are presented. Drug‐related healthcare costs within 180 days after the index date were also summarized descriptively and analyzed using a GLM with a gamma distribution and log‐link function.

The cumulative incidence of readmission was analyzed using the Kaplan–Meier method, stratified by postdischarge ONS prescription status. The first readmission was counted as an event, and patients without readmission were censored at the end of follow‐up.

## 3 Results

### 3.1 Descriptive Statistics

In total, 526 605 patients were included in the analysis, with 13 042 in the ONSd group and 513 563 in the non‐ONSd group (Figure 1). Before matching, baseline characteristics differed between the groups (Table 1). After propensity score matching, 13 042 patients remained in each group, and no significant differences in covariates were observed. During the follow‐up period, 33.2% of patients in the ONSd group received an ONS prescription, compared with only 3.8% in the non‐ONSd group (Table 2).

**TABLE 1:** The baseline characteristics of the ONSd group and the non‐ONSd group.

		Pre‐matched cohort			Matched cohort^a^		
		ONSd group	Non‐ONSd group	Standardized difference (%)	ONSd group	Non‐ONSd group	Standardized difference (%)
*N*		13 042	513 563		13 042	13 042	
Age at index month (years)	*n*	13 042	513 563	0.5	13 042	13 042	0.0
	Mean (SD)	85.3 (6.3)	82.6 (5.7)		85.3 (6.3)	85.5 (6.3)	
	Median	85.0	82.0		85.0	85.0	
	q1—q3	80.0–90.0	78.0–86.0		80.0–90.0	81.0–90.0	
	Min—max	75–107	75–111		75–107	75–110	
Gender	Female	7727 (59.2)	271 377 (52.8)	0.1	7727 (59.2)	7718 (59.2)	0.0
	Male	5315 (40.8)	242 186 (47.2)		5315 (40.8)	5324 (40.8)	
Fiscal year of index month	2017	4067 (31.2)	154 856 (30.2)	0.0	4067 (31.2)	4235 (32.5)	0.0
	2018	2851 (21.9)	112 067 (21.8)	0.0	2851 (21.9)	2804 (21.5)	0.0
	2019	2305 (17.7)	97 247 (18.9)	0.0	2305 (17.7)	2257 (17.3)	0.0
	2020	2012 (15.4)	76 116 (14.8)	0.0	2012 (15.4)	1987 (15.2)	0.0
	2021	1807 (13.9)	73 277 (14.3)	0.0	1807 (13.9)	1759 (13.5)	0.0
	2022	0 (0.0)	0 (0.0)	—	0 (0.0)	0 (0.0)	—
Admitted from emergency	No	12 733 (97.6)	504 682 (98.3)	0.0	12 733 (97.6)	12 710 (97.5)	0.0
	Yes	309 (2.4)	8880 (1.7)		309 (2.4)	332 (2.5)	
Comorbidity at index month							
Malnutrition	No	1232 (9.4)	83 347 (16.2)	−0.2	1232 (9.4)	1167 (8.9)	0.0
	Yes	11 810 (90.6)	430 215 (83.8)		11 810 (90.6)	11 875 (91.1)	
Stroke	No	8631 (66.2)	357 536 (69.6)	−0.1	8631 (66.2)	8553 (65.6)	0.0
	Yes	4411 (33.8)	156 026 (30.4)		4411 (33.8)	4489 (34.4)	
Cardiovascular disease	No	3750 (28.8)	147 725 (28.8)	0.0	3750 (28.8)	3794 (29.1)	0.0
	Yes	9292 (71.2)	365 837 (71.2)		9292 (71.2)	9248 (70.9)	
Respiratory disease	No	9535 (73.1)	414 521 (80.7)	−0.2	9535 (73.1)	9424 (72.3)	0.0
	Yes	3507 (26.9)	99 041 (19.3)		3507 (26.9)	3618 (27.7)	
Gastrointestinal disorders	No	3785 (29.0)	204 019 (39.7)	−0.2	3785 (29.0)	3696 (28.3)	0.0
	Yes	9257 (71.0)	309 543 (60.3)		9257 (71.0)	9346 (71.7)	
Psychiatric disorders	No	6911 (53.0)	398 328 (77.6)	−0.5	6911 (53.0)	6764 (51.9)	0.0
	Yes	6131 (47.0)	115 234 (22.4)		6131 (47.0)	6278 (48.1)	
Endocrine and metabolic diseases	No	11 063 (84.8)	506 471 (98.6)	−0.5	11 063 (84.8)	11 206 (85.9)	0.0
	Yes	1979 (15.2)	7091 (1.4)		1979 (15.2)	1836 (14.1)	
Musculoskeletal disorders	No	3862 (29.6)	196 714 (38.3)	−0.2	3862 (29.6)	3852 (29.5)	0.0
	Yes	9180 (70.4)	316 848 (61.7)		9180 (70.4)	9190 (70.5)	
Blood hematopoietic diseases or immune system diseases	No	9201 (70.5)	419 901 (81.8)	−0.3	9201 (70.5)	9089 (69.7)	0.0
	Yes	3841 (29.5)	93 661 (18.2)		3841 (29.5)	3953 (30.3)	
Healthcare costs (yen) within 6 months prior to index month	*n*	12 321	486 713	0.1	12 321	12 223	0.0
	Mean (SD)	901 207.9 (1 068 148.4)	783 657.2 (110 9264.3)		901 207.9 (106 8148.4)	894 667.6 (1 229 586.2)	
	Median	510 540.0	330 420.0		510 540.0	415 580.0	
	q1—q3	222 140.0–1 189 610.0	156 600.0–949 560.0		222 140.0–1 189 610.0	185 540.0–1 143 310.0	
	Min—max	590–14 261 220	140–54 094 060		590–14 261 220	250–24 891 830	
Healthcare costs of drugs (yen) within 6 months prior to index month	*n*	12 204	475 687	0.1	12 204	11 970	0.0
	Mean (SD)	162 979.5 (348 008.9)	125 211.5 (305 808.1)		162 979.5 (348 008.9)	152 253.9 (504 365.0)	
	Median	91 247.9	72 367.4		91 247.9	85 255.8	
	q1—q3	44 978.9–166 726.4	33 640.9–134 114.4		44 978.9–166 726.4	41 834.6–150 393.6	
	Min—max	17–10 452 934	2–34 490 918		17–10 452 934	17–24 457 726	
Hospital length of stay (days) within 6 months prior to index month	*n*	6726	218 522	0.0	6726	6293	−0.1
	Mean (SD)	28.2 (33.6)	29.6 (39.4)		28.2 (33.6)	31.4 (38.5)	
	Median	17.0	15.0		17.0	18.0	
	q1—q3	7.0–35.0	6.0–35.0		7.0–35.0	7.0–39.0	
	Min—max	1–184	1–244		1–184	1–213	

Abbreviations: ONS, oral nutrition supplement; SD, standard deviation.

^a^ Propensity score matching between ONSd group and non‐ONSd group was performed as the Greedy matching with a caliper of width equal to 0.2 of the standard deviation of the logit of the propensity score. The matching ratio was 1:1. Propensity scores were calculated using a logistic regression model with the covariates (age at index month, gender, fiscal year of index month, admitted from emergency, comorbidity at index month [malnutrition, stroke, cardiovascular disease, respiratory disease, gastrointestinal disorders, psychiatric disorders, endocrine and metabolic diseases, musculoskeletal disorders, blood hematopoietic diseases, or immune system diseases], healthcare costs [yen] within 6 months prior to index month, healthcare costs of drugs [yen] within 6 months prior to index month, hospital length of stay [days] within 6 months prior to index month).

**TABLE 2:** The status of ONS prescription during follow‐up period.

		Pre‐matched cohort			Matched cohort^a^		
		ONSd group	Non‐ONSd group	Standardized difference (%)	ONSd group	Non‐ONSd group	Standardized difference (%)
*N*		13 042	513 563		13 042	13 042	
The presence of ONS prescription during follow up period	No	8708 (66.8)	501 994 (97.7)	−0.9	8708 (66.8)	12 545 (96.2)	−0.8
	Yes	4334 (33.2)	11 569 (2.3)		4334 (33.2)	497 (3.8)	
Total amount of ONS prescription (g) during follow up period	*n*	4334	11 569	0.4	4334	497	0.4
	Mean (SD)	60 094.3 (79 159.6)	30 736.5 (47 699.3)		60 094.3 (79 159.6)	35 923.2 (52 029.8)	
	Median	29 750.0	12 000.0		29 750.0	15 000.0	
	q1—q3	10 500.0–80 350.0	4375.0–35 750.0		10 500.0–80 350.0	5600.0–42 875.0	
	Min—max	25–1 335 600	3–611 100		25–1 335 600	250–449 400	
Frequency of ONS prescription (/day) during follow up period	*n*	4334	11 569	0.7	4334	497	0.5
	Mean (SD)	0.0185 (0.0132)	0.0106 (0.0106)		0.0185 (0.0132)	0.0127 (0.0121)	
	Median	0.0164	0.0072		0.0164	0.0093	
	q1—q3	0.0082–0.0265	0.0026–0.0163		0.0082–0.0265	0.0039–0.0192	
	Min—max	0.001–0.178	0.001–0.323		0.001–0.178	0.001–0.115	
Number of ONS prescription during follow up period	*n*	4334	11 569	0.4	4334	497	0.3
	Mean (SD)	6.8 (7.7)	4.3 (5.3)		6.8 (7.7)	5.0 (6.4)	
	Median	4.0	2.0		4.0	3.0	
	q1—q3	2.0–9.0	1.0–5.0		2.0–9.0	1.0–6.0	
	Min—max	1–93	1–81		1–93	1–72	
Total amount of ONS prescription (g) during follow up period	0	8708 (66.8)	501 994 (97.7)	−0.9	8708 (66.8)	12 545 (96.2)	−0.8
	0< to ≤ 1000	20 (0.2)	287 (0.1)	0.0	20 (0.2)	9 (0.1)	0.0
	1000< to ≤ 7000	717 (5.5)	4079 (0.8)	0.3	717 (5.5)	147 (1.1)	0.2
	7000< to ≤ 30 000	1463 (11.2)	3950 (0.8)	0.5	1463 (11.2)	185 (1.4)	0.4
	30 000<	2134 (16.4)	3253 (0.6)	0.6	2134 (16.4)	156 (1.2)	0.6
Frequency of ONS prescription during follow up period	No prescription	8708 (66.8)	501 994 (97.7)	−0.9	8708 (66.8)	12 545 (96.2)	−0.8
	Not more than once in 90 days	1461 (11.2)	7570 (1.5)	0.4	1461 (11.2)	290 (2.2)	0.4
	Not more than once in 30 days	2628 (20.2)	3768 (0.7)	0.7	2628 (20.2)	194 (1.5)	0.6
	Not more than once in 14 days	215 (1.6)	214 (0.0)	0.2	215 (1.6)	10 (0.1)	0.2
	Not more than once in 7 days	27 (0.2)	16 (0.0)	0.1	27 (0.2)	3 (0.0)	0.1
	More than once in 7 days	3 (0.0)	1 (0.0)	0.0	3 (0.0)	0 (0.0)	0.0
Number of ONS prescription during follow up period	0	8708 (66.8)	501 994 (97.7)	−0.9	8708 (66.8)	12 545 (96.2)	−0.8
	1	1006 (7.7)	4452 (0.9)	0.3	1006 (7.7)	157 (1.2)	0.3
	2	634 (4.9)	1891 (0.4)	0.3	634 (4.9)	83 (0.6)	0.3
	3	443 (3.4)	1149 (0.2)	0.2	443 (3.4)	57 (0.4)	0.2
	4–6	789 (6.0)	1773 (0.3)	0.3	789 (6.0)	81 (0.6)	0.3
	7–9	389 (3.0)	874 (0.2)	0.2	389 (3.0)	43 (0.3)	0.2
	10≤	1073 (8.2)	1430 (0.3)	0.4	1073 (8.2)	76 (0.6)	0.4

Abbreviations: ONS, oral nutrition supplement; SD, standard deviation.

^a^ Propensity score matching between ONSd group and non‐ONSd group was performed as the Greedy matching with a caliper of width equal to 0.2 of the standard deviation of the logit of the propensity score. The matching ratio was 1:1. Propensity scores were calculated using a logistic regression model with the covariates (age at index month, gender, fiscal year of index month, admitted from emergency, comorbidity at index month [malnutrition, stroke, cardiovascular disease, respiratory disease, gastrointestinal disorders, psychiatric disorders, endocrine and metabolic diseases, musculoskeletal disorders, blood hematopoietic diseases or immune system diseases], healthcare costs [yen] within 6 months prior to the index month, healthcare costs of drugs [yen] within 6 months prior to the index month, hospital length of stay [days] within 6 months prior to the index month).

### 3.2 Primary Analysis

**FIGURE 2:**
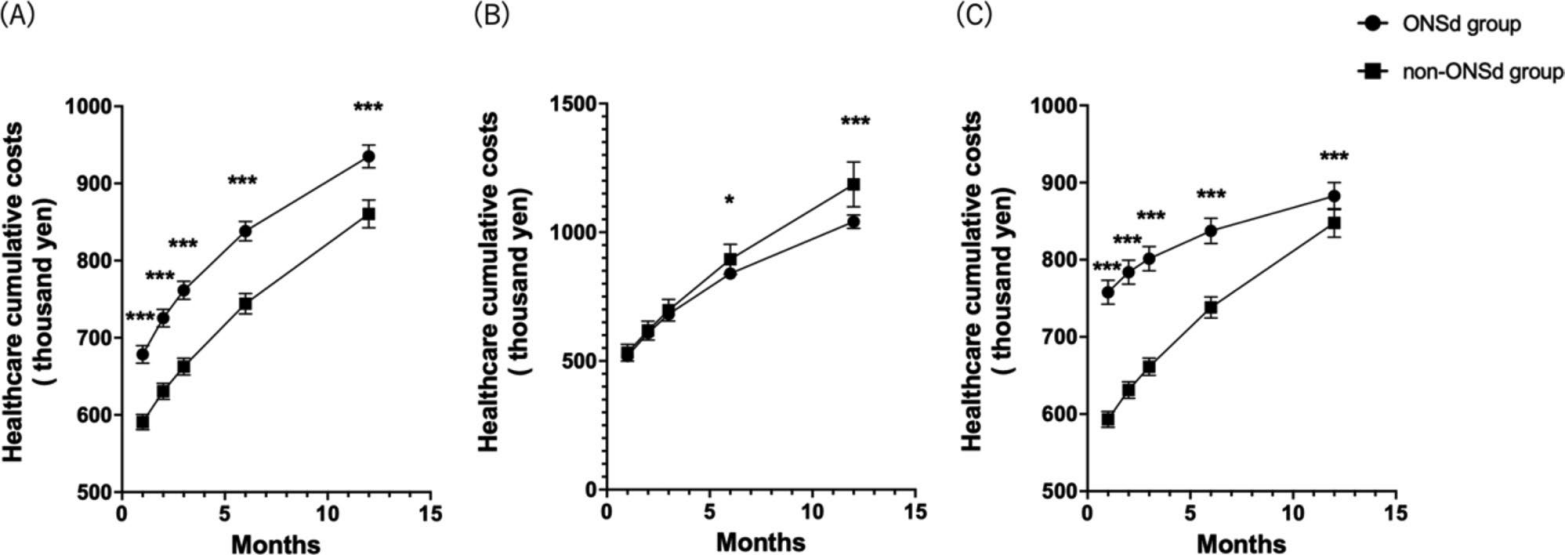
Cumulative healthcare costs after hospital discharge. Cumulative healthcare costs (in yen) are shown for the ONSd group and non‐ONSd group, with error bars representing 95% confidence intervals. (A) whole cohort; (B) patients who received ONS prescriptions during follow‐up; (C) patients who received no ONS prescriptions during follow‐up. Statistical significance is indicated as follows: *adjusted *p* < 0.05, ***adjusted *p* < 0.001. Statistical significance in the figure is based on Benjamini–Hochberg–adjusted *p* values.

At 30, 60, 90, 180, and 360 days postdischarge, healthcare costs were consistently higher in the ONSd group than in the non‐ONSd group (adjusted *p* < 0.001). At 360 days, the mean healthcare cost was 935 092 yen in the ONSd group compared with 860 588 yen in the non‐ONSd group (Figure 2A, Table S1).

Among patients who received ONS prescriptions during follow‐up, those in the ONSd group incurred significantly lower healthcare costs than those in the non‐ONSd group at 180 days (839 623 vs. 895 002 yen, adjusted *p* < 0.05) and at 360 days (1 040 830 vs. 1 185 919 yen, adjusted *p* < 0.001) (Figure 2B, Table S2). Conversely, among patients who did not receive ONS prescriptions during follow‐up, healthcare costs were significantly higher in the ONSd group than in the non‐ONSd group at all corresponding time points (adjusted *p* < 0.001) (Figure 2C, Table S3). In the IPTW‐weighted sensitivity analysis, similar patterns were observed: healthcare costs at each time point were consistently higher in the ONSd group in the overall cohort (adjusted *p* < 0.001). Among patients who received ONS prescriptions during follow‐up, the ONSd group showed lower cumulative healthcare costs at 360 days (1 082 959.2 yen vs. 1 163 425.3 yen, adjusted *p* < 0.001), whereas among those without ONS prescriptions during follow‐up, costs remained higher in the ONSd group across all time points (Table S4).

To further examine whether these cost differences reflected treatment adherence, we conducted stratified analyses using the amount and frequency of ONS prescriptions during follow‐up as proxies for adherence. Across both measures, patients in the ONSd group generally demonstrated lower cumulative healthcare costs than those in the non‐ONSd group, except in the lowest ONS prescription stratum (< 1000 g total ONS during follow‐up), where the difference was not significant (Table S5).

### 3.3 Secondary Analysis

#### 3.3.1 Healthcare Use

At 360 days postdischarge, the mean LOS for readmission was 27.8 and 22.6 days in the ONSd and non‐ONSd groups, respectively, with a significantly longer duration in the ONSd group (*p* < 0.001) (Table S6). Conversely, the number of outpatient physician visits was significantly higher in the non‐ONSd group than in the ONSd group (mean visits: 5.1 vs. 4.3, *p* < 0.001) (Table S6). There was no statistically significant difference in emergency room visits between the ONSd and non‐ONSd groups, and most participants did not require any emergency visits.

#### 3.3.2 Drug‐Related Healthcare Costs

At 180 days postdischarge, the mean drug‐related healthcare cost was 124 546.5 yen in the ONSd group and 112 010.7 yen in the non‐ONSd group (Table S7). However, after adjusting for ONS prescription during the follow‐up period, this difference was not significant.

#### 3.3.3 Cumulative Incidence of Readmission

Throughout the follow‐up period, the cumulative incidence of readmission was consistently higher in the ONSd group than in the non‐ONSd group. At 360 days, the cumulative incidence was 59.7% in the ONSd group and 44.4% in the non‐ONSd group (Figure S1). Similar patterns were observed in stratified analyses based on ONS prescription status during follow‐up.

## 4 Discussion

This study provides new evidence on the relationship between ONS prescription at hospital discharge and healthcare costs among older adults in Japan, using a large‐scale claims database.

We hypothesized that ONS prescribed at discharge would improve nutritional status, thereby be associated with better clinical outcomes and lower postdischarge healthcare costs. However, in the overall matched cohort, our results did not support this hypothesis. Total costs over 360 days were higher in the ONSd group, which also had longer LOS and higher readmission rates. In the cost breakdown at 180 days, drug‐related expenditures—including the cost of ONS—were only slightly higher in the ONSd group (124 546.5 yen vs. 112 010.7 yen) (Table S7). In contrast, inpatient and outpatient costs combined showed a much larger difference (713 574.7 yen vs. 632 077.9 yen).

Secondary outcomes were consistent with these patterns. The non‐ONSd group had slightly more outpatient visits, whereas the ONSd group had longer LOS during readmissions (Table S6). The cumulative incidence of readmission was also higher in the ONSd group throughout follow‐up (Figure S1). These findings suggest that inpatient costs, rather than outpatient or drug‐related costs, largely contributed to the overall cost differences.

Previous studies have indicated that ONS use can reduce healthcare costs associated with malnutrition by shortening hospital stays, lowering costs per care episode, and reducing relapse rates [15, 17, 19, 20, 26, 27]. In contrast, our real‐world, retrospective analysis in the Japanese healthcare setting revealed higher direct costs and a higher readmission rate. Several factors may explain this discrepancy.

First, previous studies provided ONS during hospitalization under controlled conditions, whereas our study evaluated ONS use at home postdischarge. Without structured postdischarge support, adherence in real‐world practice is likely lower than that in clinical trials, potentially diminishing clinical benefits while still incurring product costs. Second, the mean age of our population was approximately 85 years—considerably higher than in previous studies, which typically included participants in their 60s or 70s. Additionally, higher adherence is strongly associated with better nutritional outcomes [28]. However, advanced age may reduce adherence due to dysphagia, cognitive impairment, and decreased appetite. Age‐related physiological changes may also limit the effectiveness of nutritional interventions. Similarly, studies involving very old adults have reported limited intervention effects and difficulty maintaining adherence [29, 30]. Third, despite propensity score matching, residual confounding may have persisted. Clinicians may have been more likely to prescribe ONS at discharge to frailer patients or those at higher nutritional risk, leading to greater healthcare utilization independent of ONS use, unlike in randomized controlled trials.

Our subgroup analysis showed that among patients who received ONS prescriptions during follow‐up (indicating at least some degree of use and adherence, as reflected in the prescription patterns in Table 2), the ONSd group incurred lower healthcare costs at both 180 and 360 days than the non‐ONSd group. A similar finding was observed in the IPTW‐weighted sensitivity analysis, in which the ONSd group had lower 360‐day costs. Moreover, the cost difference widened progressively at 30, 60, 90, 180, and 360 days postdischarge, suggesting an association between cumulative and sustained ONS use and the observed long‐term cost differences.

To address the apparent contradiction between these findings and the higher overall costs in the full cohort, we considered whether this discrepancy might be explained by adherence‐related factors. Because receiving an ONS prescription after discharge may itself indicate higher adherence, we performed stratified analyses using total ONS prescription amount and prescription frequency as adherence proxies. Notably, even after stratification by these proxies, the ONSd group consistently showed lower cumulative healthcare costs across nearly all strata (Table S5). These findings suggest that, although residual confounding cannot be excluded, the benefits observed in patients who continued ONS for a certain period are unlikely attributable to adherence bias alone and may reflect the advantage of early ONS initiation at discharge.

In contrast, among patients who were not prescribed ONS during follow‐up, the ONSd group had substantially higher healthcare costs in the early postdischarge period; however, this difference gradually diminished over the 12‐month period (Figure 2C). This convergence may partially reflect the higher early readmission rate in the ONSd group (Figure S1). Patients discharged with nutritional concerns may have received more proactive or lower‐threshold inpatient care soon after discharge. Over time, this early medical attention could have helped prevent more severe or costly long‐term complications, resulting in a more stable healthcare expenditure during later follow‐up. Although the EFFORT trial focused primarily on short‐term outcomes, a secondary analysis showed that inpatient‐only nutritional support did not provide sustained benefits at 6 months, highlighting the limitations of discontinuing nutritional care at discharge [31]. Collectively, these findings indicate the importance of timely initiation and continued nutritional support.

Patients in the ONSd group experienced longer readmission stays and higher readmission rates but fewer outpatient visits than those in the non‐ONSd group. Despite propensity score matching, these trends may reflect residual confounding factors such as mobility limitations, delayed recognition of clinical deterioration, or higher underlying morbidity. However, confounding alone may not fully account for these patterns. They may also suggest that nutritional intervention at discharge, when not paired with structured postdischarge monitoring, may be insufficient to prevent deterioration. Therefore, ONS prescription at discharge alone may be inadequate and that coordinated postdischarge follow‐up may be necessary to reduce recurrent hospitalizations and severe exacerbations requiring prolonged inpatient care.

Despite these findings, several limitations should be acknowledged. First, ONS prescriptions recorded in the KDB were used as a proxy for actual intake. Because ONS regimen adherence could not be directly assessed, the observed association between continued ONS use and lower healthcare costs should be interpreted with caution. Although prescription amount and frequency can serve as indirect indicators of adherence (Table 2), they do not necessarily reflect true consumption. Future studies with detailed adherence data and objective nutritional assessments are needed to validate these findings. Second, considering the observational design, unmeasured confounding may persist despite propensity score matching. The KDB lacks information on disease severity, laboratory findings, and key clinical characteristics such as nutritional status, frailty, functional ability, and social support, limiting the ability to fully account for potential confounders or effect modifiers. Additionally, some patients classified in the non‐ONSd group initiated ONS during follow‐up. Because grouping was based on prescription status at discharge, these patients remained in the non‐ONSd group. Although this approach reflects the clinical decision point at discharge, it may have introduced residual confounding. Third, we could not distinguish between patients newly prescribed ONS at discharge and those who had been using ONS before hospitalization. Pre‐existing ONS use may reflect differences in baseline health status or prognosis, and residual confounding from this factor cannot be excluded. Fourth, the KDB does not include baseline nutritional indicators such as body mass index or recent weight change. Therefore, we could not evaluate patients' nutritional status at discharge, limiting interpretation regarding the appropriateness and potential effectiveness of ONS prescription. Fifth, the KDB captures only services reimbursed through medical insurance; thus, workers' compensation, automobile liability insurance, and non‐reimbursed services, such as normal childbirth, cosmetic procedures, and advanced medical care, are not included. Finally, although the total sample size exceeded 500 000 patients, the ONSd group (≈13 000 patients) was relatively small. Moreover, given the advanced mean age (~85 years), our findings primarily reflect outcomes in a super‐aged population with prevalent multimorbidity and frailty. While caution is needed when generalizing these results to younger or different populations, they provide important insights into nutritional management in rapidly aging societies.

Overall, our findings highlight the complexity of implementing nutritional interventions in very old adults in real‐world settings. Effective ONS use may require timely prescription and coordinated strategies to support adherence and continuous monitoring. Future research should examine integrated care models that combine nutritional support with structured postdischarge follow‐up to optimize clinical and economic outcomes.

## Author Contributions

Study conceptualization: T.H., M.Ya., and S.O. Study design: M.M., T.H., M.Ya., and S.O. Data acquisition and data analysis: M.M., T.H., and M.Ya. Interpretation of data: M.M., T.H., M.Yu., Y.K., M.Ya., and S.O. Drafting the manuscript and revising the manuscript: M.M. and T.H.

## Funding

The authors have nothing to report.

## Ethics Statement

The study's protocol was approved by the Ethics Committee of the Faculty of Medicine, The University of Tokyo.

## Conflicts of Interest

S.O. received research funding from Abbott Japan LLC. The other authors declare no conflicts of interest.

## Supporting information


**Data S1:** Supporting information.

## Data Availability

The data that support the findings of this study are available from the corresponding author upon reasonable request.

## References

[1] F. Bellanti , A. Lo Buglio , S. Quiete , and G. Vendemiale , “Malnutrition in Hospitalized Old Patients: Screening and Diagnosis, Clinical Outcomes, and Management,” Nutrients 14, no. 4 (2022): 910. 35215559 10.3390/nu14040910 PMC8880030

[2] L. J. Curtis , P. Bernier , K. Jeejeebhoy , et al., “Costs of Hospital Malnutrition,” Clinical Nutrition 36, no. 5 (2017): 1391–1396. 27765524 10.1016/j.clnu.2016.09.009

[3] Y. Fukuta , S. Arizono , N. Funaguchi , et al., “Relationship Between Poor Nutritional Status and Functional Independence Measure Scores, Physical Functioning in Older Patients With Pneumonia,” Therapeutic Research 45, no. 9 (2024): 601–610.

[4] L. Botero , M. D. Banks , E. H. Gordon , J. Bauer , and A. M. Young , “Incidence and Outcomes of In‐Hospital Nutritional Decline: A Prospective Observational Cohort Study in Adult Patients,” Clinical Nutrition 43, no. 5 (2024): 1057–1064. 38569329 10.1016/j.clnu.2024.03.014

[5] R. Burgos , C. Joaquín , C. Blay , and C. Vaqué , “Disease‐Related Malnutrition in Hospitalized Chronic Patients With Complex Needs,” Clinical Nutrition 39, no. 5 (2020): 1447–1453. 31256806 10.1016/j.clnu.2019.06.006

[6] J. Schilp , H. M. Kruizenga , H. A. Wijnhoven , et al., “High Prevalence of Undernutrition in Dutch Community‐Dwelling Older Individuals,” Nutrition 28, no. 11–12 (2012): 1151–1156. 22749873 10.1016/j.nut.2012.02.016

[7] M. Díaz , A. P. de Arenas Larriva , G. Molina‐Recio , R. Moreno‐Rojas , and M. de la Iglesia , “Study of the Nutritional Status of Patients Over 65 Years Included in the Home Care Program in an Urban Population,” Atencion Primaria 50, no. 2 (2017): 88–95. 28595900 10.1016/j.aprim.2017.02.006 PMC6837150

[8] E. Cereda , C. Pedrolli , C. Klersy , et al., “Nutritional Status in Older Persons According to Healthcare Setting: A Systematic Review and Meta‐Analysis of Prevalence Data Using MNA,” Clinical Nutrition 35, no. 6 (2016): 1282–1290. 27086194 10.1016/j.clnu.2016.03.008

[9] S. Ogawa , T. Hosoi , M. Akishita , and A. Igarashi , “Malnutrition‐Related Health Care Cost in Japan: An Analysis of Health Insurance Claims Data,” Asia‐Pacific Journal of Public Health 31, no. 7 (2019): 594–602. 31537120 10.1177/1010539519874946

[10] M. Elia , The Cost of Malnutrition in England and Potential Cost Savings From Nutritional Interventions (Full Report) (Malnutrition Action Group of BAPEN and National Institute for Health Research Southampton Biomedical Research Centre, 2015).

[11] S. Goates , K. Du , C. A. Braunschweig , and M. B. Arensberg , “Economic Burden of Disease‐Associated Malnutrition at the State Level,” PLoS One 11, no. 9 (2016): e0161833. 27655372 10.1371/journal.pone.0161833 PMC5031313

[12] A. Inotai , M. Nuijten , E. Roth , R. Hegazi , and Z. Kaló , “Modelling the Burden of Disease Associated Malnutrition,” E‐SPEN Journal 7, no. 5 (2012): e196–e204.

[13] M. T. Linthicum , J. Thornton Snider , R. Vaithianathan , et al., “Economic Burden of Disease‐Associated Malnutrition in China,” Asia‐Pacific Journal of Public Health 27, no. 4 (2015): 407–417. 25301845 10.1177/1010539514552702

[14] K. Freijer , S. S. Tan , M. A. Koopmanschap , J. M. Meijers , R. J. Halfens , and M. J. Nuijten , “The Economic Costs of Disease Related Malnutrition,” Clinical Nutrition 32, no. 1 (2013): 136–141. 22789931 10.1016/j.clnu.2012.06.009

[15] T. J. Philipson , J. T. Snider , D. N. Lakdawalla , B. Stryckman , and D. P. Goldman , “Impact of Oral Nutritional Supplementation on Hospital Outcomes,” American Journal of Managed Care 19, no. 2 (2013): 121–128. 23448109

[16] D. N. Lakdawalla , J. T. Snider , and D. J. Perlroth , “Can Oral Nutritional Supplements Improve Medicare Patient Outcomes in the Hospital?,” Forum for Health Economics and Policy 17 (2014): 131–151. 31419880 10.1515/fhep-2014-0011

[17] Y. Zhong , J. T. Cohen , S. Goates , M. Luo , J. Nelson , and P. J. Neumann , “The Cost‐Effectiveness of Oral Nutrition Supplementation for Malnourished Older Hospital Patients,” Applied Health Economics and Health Policy 15 (2017): 75–83. 27492419 10.1007/s40258-016-0269-7 PMC5253145

[18] M. Elia , E. L. Parsons , A. L. Cawood , T. R. Smith , and R. J. Stratton , “Cost‐Effectiveness of Oral Nutritional Supplements in Older Malnourished Care Home Residents,” Clinical Nutrition 37, no. 2 (2018): 651–658. 28279548 10.1016/j.clnu.2017.02.008

[19] A. Kapedanovska Nestorovska and Z. Sterjev , “Cost‐Effectiveness of Oral Nutritional Supplements in Malnourished or at Risk of Disease‐Related Malnutrition Cancer Patients in North Macedonia,” ClinicoEconomics and Outcomes Research 17 (2025): 265–276. 40165978 10.2147/CEOR.S504094 PMC11955403

[20] S. Wang , J. Shafrin , K. W. Kerr , and P. Schuetz , “Health Economic Value of Postacute Oral Nutritional Supplementation in Older Adult Medical Patients at Risk for Malnutrition: A US‐Based Modelling Approach,” BMJ Open 14, no. 11 (2024): e086787. 10.1136/bmjopen-2024-086787 PMC11574475 39551592

[21] K. H. Thomson , S. Rice , O. Arisa , et al., “Effectiveness and Cost‐Effectiveness of Oral Nutritional Supplements in Frail Older People Who Are Malnourished or at Risk of Malnutrition: A Systematic Review and Meta‐Analysis,” Lancet Healthy Longevity 3, no. 10 (2022): e654–e666. 36116457 10.1016/S2666-7568(22)00171-4

[22] E. Cereda , R. Pisati , M. Rondanelli , and R. Caccialanza , “Whey Protein, Leucine‐ and Vitamin‐D‐Enriched Oral Nutritional Supplementation for the Treatment of Sarcopenia,” Nutrients 14, no. 7 (2022): 1524. 35406137 10.3390/nu14071524 PMC9003251

[23] P. Schuetz , R. Fehr , V. Baechli , et al., “Individualised Nutritional Support in Medical Inpatients at Nutritional Risk: A Randomised Clinical Trial,” Lancet 393, no. 10188 (2019): 2312–2321. 31030981 10.1016/S0140-6736(18)32776-4

[24] P. Schuetz , S. Sulo , S. Walzer , et al., “Economic Evaluation of Individualized Nutritional Support in Medical Inpatients: Secondary Analysis of the EFFORT Trial,” Clinical Nutrition 39, no. 11 (2020): 3361–3368. 32147200 10.1016/j.clnu.2020.02.023

[25] N. Ikegami , B.‐K. Yoo , H. Hashimoto , et al., “Japanese Universal Health Coverage: Evolution, Achievements, and Challenges,” Lancet 378, no. 9796 (2011): 1106–1115. 21885107 10.1016/S0140-6736(11)60828-3

[26] R. J. Stratton , X. Hebuterne , and M. Elia , “A Systematic Review and Meta‐Analysis of the Impact of Oral Nutritional Supplements on Hospital Readmissions,” Ageing Research Reviews 12, no. 4 (2013): 884–897. 23891685 10.1016/j.arr.2013.07.002

[27] K. Sriram , S. Sulo , G. VanDerBosch , et al., “A Comprehensive Nutrition‐Focused Quality Improvement Program Reduces 30‐Day Readmissions and Length of Stay in Hospitalized Patients,” Journal of Parenteral and Enteral Nutrition 41, no. 3 (2017): 384–391. 27923890 10.1177/0148607116681468

[28] D. A. Chavarro‐Carvajal , A. M. Ayala , L. C. Venegas‐Sanabria , et al., “Use of a Nutrition‐Focused Quality Improvement Program for Community‐Living Older Adults at Malnutrition Risk Is Associated With Better Nutritional Outcomes,” Clinical Nutrition ESPEN 48 (2022): 291–297. 35331504 10.1016/j.clnesp.2022.01.032

[29] R. Price , F. Daly , C. R. Pennington , and M. E. McMurdo , “Nutritional Supplementation of Very Old People at Hospital Discharge Increases Muscle Strength: A Randomised Controlled Trial,” Gerontology 51, no. 3 (2005): 179–185. 15832045 10.1159/000083991

[30] M. D. Miller , M. Crotty , C. Whitehead , E. Bannerman , and L. A. Daniels , “Nutritional Supplementation and Resistance Training in Nutritionally at Risk Older Adults Following Lower Limb Fracture: A Randomized Controlled Trial,” Clinical Rehabilitation 20, no. 4 (2006): 311–323. 16719029 10.1191/0269215506cr942oa

[31] N. Kaegi‐Braun , P. Tribolet , F. Gomes , et al., “Six‐Month Outcomes After Individualized Nutritional Support During the Hospital Stay in Medical Patients at Nutritional Risk: Secondary Analysis of a Prospective Randomized Trial,” Clinical Nutrition 40, no. 3 (2021): 812–819. 32919819 10.1016/j.clnu.2020.08.019

