# Supplementary material for: Impact of Oral Nutritional Supplementation at Hospital Discharge on Healthcare Costs in Older Adults: A Retrospective Analysis of Japanese Claims Data
Source: Geriatr Gerontol Int. 2025 Dec 26;26(1):e70288. doi: 10.1111/ggi.70288 (PMC12803869; doi:10.1111/ggi.70288)
Supplement: Supplementary file 1 — Data S1: Supporting information. [file GGI-26-0-s001.docx]

Supplementary Table 1 Cumulative healthcare costs (in yen) over 12 months after discharge

| **Supplementary Table 1.1 Healthcare costs within 1-month** | | | |  |  |  |  |
| --- | --- | --- | --- | --- | --- | --- | --- |
|  |  |  |  |  |  |  |  |
|  |  |  | PS matched cohort | | | |  |
|  |  |  | ONSd group | Non-ONSd group | Ratio (95%CI) | P-value | Adjusted  P-value |
|  | Healthcare costs (yen) | n | 13042 | 13042 | 0.871  (0.855 - 0.887) | <0.001 | <0.001 |
|  |  | mean (SD) | 678525.0 (660114.9) | 590835.5 (576275.6) |  |  |  |
|  |  | median | 507465.0 | 438875.0 |  |  |  |
|  |  | q1 - q3 | 296020.0 - 831200.0 | 244330.0 - 750770.0 |  |  |  |
|  |  | min - max | 1800 - 11801720 | 3840 - 10246690 |  |  |  |
|  |  |  |  |  |  |  |  |
| **Supplementary Table 1.2 Healthcare costs within 2-months** | | | |  |  |  |  |
|  |  |  |  |  |  |  |  |
|  |  |  | PS matched cohort | | | |  |
|  |  |  | ONSd group | Non-ONSd group | Ratio (95%CI) | P-value | Adjusted  P-value |
|  | Healthcare costs (yen) | n | 13042 | 13042 | 0.869  (0.854 - 0.885) | <0.001 | <0.001 |
|  |  | mean (SD) | 725564.0 (667632.6) | 630622.6 (601371.4) |  |  |  |
|  |  | median | 553190.0 | 477735.0 |  |  |  |
|  |  | q1 - q3 | 335100.0 - 890640.0 | 274140.0 - 797060.0 |  |  |  |
|  |  | min - max | 6790 - 11989870 | 6280 - 12132090 |  |  |  |
|  |  |  |  |  |  |  |  |
| **Supplementary Table 1.3 Healthcare costs within 3-months** | | | |  |  |  |  |
|  |  |  |  |  |  |  |  |
|  |  |  | PS matched cohort | | | |  |
|  |  |  | ONSd group | Non-ONSd group | Ratio (95%CI) | P-value | Adjusted  P-value |
|  | Healthcare costs (yen) | n | 13042 | 13042 | 0.870  (0.855 - 0.886) | <0.001 | <0.001 |
|  |  | mean (SD) | 761450.0 (681321.1) | 662643.6 (629036.8) |  |  |  |
|  |  | median | 588915.0 | 508885.0 |  |  |  |
|  |  | q1 - q3 | 361040.0 - 930430.0 | 296670.0 - 829220.0 |  |  |  |
|  |  | min - max | 6790 - 11989870 | 6280 - 13909180 |  |  |  |
|  |  |  |  |  |  |  |  |
| **Supplementary Table 3.4 Healthcare costs within 6-months** | | | |  |  |  |  |
|  |  |  |  |  |  |  |  |
|  |  |  | PS matched cohort | | | |  |
|  |  |  | ONSd group | Non-ONSd group | Ratio (95%CI) | P-value | Adjusted  P-value |
|  | Healthcare costs (yen) | n | 13042 | 13042 | 0.888  (0.872 - 0.904) | <0.001 | <0.001 |
|  |  | mean (SD) | 838121.2 (732631.6) | 744088.6 (776190.5) |  |  |  |
|  |  | median | 663210.0 | 575775.0 |  |  |  |
|  |  | q1 - q3 | 409440.0 - 1016760.0 | 345910.0 - 906930.0 |  |  |  |
|  |  | min - max | 6790 - 11989870 | 7220 - 28389910 |  |  |  |
|  |  |  |  |  |  |  |  |
| **Supplementary Table 3.5 Healthcare costs within 12-months** | | | |  |  |  |  |
|  |  |  |  |  |  |  |  |
|  |  |  | PS matched cohort | | | |  |
|  |  |  | ONSd group | Non-ONSd group | Ratio (95%CI) | P-value | Adjusted  P-value |
|  | Healthcare costs (yen) | n | 13042 | 13042 | 0.920  (0.904 - 0.937) | <0.001 | <0.001 |
|  |  | mean (SD) | 935091.5 (854739.6) | 860588.3 (1055795.8) |  |  |  |
|  |  | median | 731110.0 | 663960.0 |  |  |  |
|  |  | q1 - q3 | 449570.0 - 1124490.0 | 392460.0 - 1025770.0 |  |  |  |
|  |  | min - max | 6790 - 12507610 | 7220 - 52708680 |  |  |  |

*Adjusted P values correspond to Benjamini–Hochberg–adjusted P values applied for multiple-comparison control.

Supplementary Table 2 Cumulative healthcare costs (in yen) over 12 months after discharge among patients who were prescribed ONS during follow-up

| **Supplementary Table 2.1 Healthcare costs within 1-month (Patients with ONS during study period)** | | | | | |  |  |
| --- | --- | --- | --- | --- | --- | --- | --- |
|  |  |  |  |  |  |  |  |
|  |  |  | PS matched cohort | | | | |
|  |  |  | ONSd group | Non-ONSd group | Ratio (95%CI) | P-value | Adjusted P-value |
|  | Healthcare costs (yen) | n | 4334 | 497 | 1.025 (0.961 - 1.092) | 0.456 | 0.489 |
|  |  | mean (SD) | 519223.9 (417726.2) | 532003.0 (371012.8) |  |  |  |
|  |  | median | 416900.0 | 463920.0 |  |  |  |
|  |  | q1 - q3 | 248560.0 - 654500.0 | 246530.0 - 695690.0 |  |  |  |
|  |  | min - max | 1800 - 5076020 | 30200 - 2307770 |  |  |  |
|  |  |  |  |  |  |  |  |
| **Supplementary Table 2.2 Healthcare costs within 2-months (Patients with ONS during study period)** | | | | | |  |  |
|  |  |  |  |  |  |  |  |
|  |  |  | PS matched cohort | | | | |
|  |  |  | ONSd group | Non-ONSd group | Ratio (95%CI) | P-value | Adjusted P-value |
|  | Healthcare costs (yen) | n | 4334 | 497 | 1.015 (0.956 - 1.079) | 0.625 | 0.625 |
|  |  | mean (SD) | 608527.8 (460872.8) | 617812.2 (414612.7) |  |  |  |
|  |  | median | 492725.0 | 522310.0 |  |  |  |
|  |  | q1 - q3 | 311030.0 - 753170.0 | 307560.0 - 821690.0 |  |  |  |
|  |  | min - max | 18210 - 5360630 | 48800 - 2323000 |  |  |  |
|  |  |  |  |  |  |  |  |
| **Supplementary Table 2.3 Healthcare costs within 3-months (Patients with ONS during study period)** | | | | | |  |  |
|  |  |  |  |  |  |  |  |
|  |  |  | PS matched cohort | | | | |
|  |  |  | ONSd group | Non-ONSd group | Ratio (95%CI) | P-value | Adjusted P-value |
|  | Healthcare costs (yen) | n | 4334 | 497 | 1.023 (0.964 - 1.085) | 0.450 | 0.519 |
|  |  | mean (SD) | 681361.3 (504670.2) | 697089.6 (476790.6) |  |  |  |
|  |  | median | 556495.0 | 575080.0 |  |  |  |
|  |  | q1 - q3 | 358260.0 - 842770.0 | 372600.0 - 900670.0 |  |  |  |
|  |  | min - max | 26960 - 5658160 | 53150 - 3574160 |  |  |  |
|  |  |  |  |  |  |  |  |
| **Supplementary Table 2.4 Healthcare costs within 6-months (Patients with ONS during study period)** | | | | | |  |  |
|  |  |  |  |  |  |  |  |
|  |  |  | PS matched cohort | | | | |
|  |  |  | ONSd group | Non-ONSd group | Ratio (95%CI) | P-value | Adjusted P-value |
|  | Healthcare costs (yen) | n | 4334 | 497 | 1.066 (1.006 - 1.130) | 0.032 | 0.040 |
|  |  | mean (SD) | 839623.0 (630309.9) | 895001.5 (657666.4) |  |  |  |
|  |  | median | 688545.0 | 728250.0 |  |  |  |
|  |  | q1 - q3 | 457110.0 - 1009000.0 | 476650.0 - 1065900.0 |  |  |  |
|  |  | min - max | 33720 - 7106360 | 67990 - 4475210 |  |  |  |
|  |  |  |  |  |  |  |  |
| **Supplementary Table 2.5 Healthcare costs within 12-months (Patients with ONS during study period)** | | | | | |  |  |
|  |  |  |  |  |  |  |  |
|  |  |  | PS matched cohort | | | | |
|  |  |  | ONSd group | Non-ONSd group | Ratio (95%CI) | P-value | Adjusted P-value |
|  | Healthcare costs (yen) | n | 4334 | 497 | 1.139 (1.072 - 1.211) | <0.001 | <0.001 |
|  |  | mean (SD) | 1040829.7 (882059.7) | 1185919.4 (987373.3) |  |  |  |
|  |  | median | 829060.0 | 957950.0 |  |  |  |
|  |  | q1 - q3 | 548470.0 - 1226390.0 | 604940.0 - 1378240.0 |  |  |  |
|  |  | min - max | 34960 - 12507610 | 79590 - 7223880 |  |  |  |

*Adjusted P values correspond to Benjamini–Hochberg–adjusted P values applied for multiple-comparison control.

Supplementary Table 3 Cumulative healthcare costs (in yen) over 12 months after discharge among patients who were not prescribed ONS during follow-up

| **Supplementary Table 3.1 Healthcare costs within 1-month (Patients without ONS during study period)** | | | | | |  |  |
| --- | --- | --- | --- | --- | --- | --- | --- |
|  |  |  |  |  |  |  |  |
|  |  |  | PS matched cohort | | | | |
|  |  |  | ONSd group | Non-ONSd group | Ratio (95%CI) | P-value | Adjusted P-value |
|  | Healthcare costs (yen) | n | 8708 | 12545 | 0.783 (0.766 - 0.800) | <0.001 | <0.001 |
|  |  | mean (SD) | 757809.7 (739524.5) | 593166.3 (582808.6) |  |  |  |
|  |  | median | 567370.0 | 438040.0 |  |  |  |
|  |  | q1 - q3 | 326610.0 - 936180.0 | 244140.0 - 753840.0 |  |  |  |
|  |  | min - max | 6790 - 11801720 | 3840 - 10246690 |  |  |  |
|  |  |  |  |  |  |  |  |
| **Supplementary Table 3.2 Healthcare costs within 2-months (Patients without ONS during study period)** | | | | | |  |  |
|  |  |  |  |  |  |  |  |
|  |  |  | PS matched cohort | | | | |
|  |  |  | ONSd group | Non-ONSd group | Ratio (95%CI) | P-value | Adjusted P-value |
|  | Healthcare costs (yen) | n | 8708 | 12545 | 0.805 (0.789 - 0.822) | <0.001 | <0.001 |
|  |  | mean (SD) | 783813.4 (742757.3) | 631130.1 (607595.5) |  |  |  |
|  |  | median | 593865.0 | 476340.0 |  |  |  |
|  |  | q1 - q3 | 349500.0 - 969910.0 | 272740.0 - 796370.0 |  |  |  |
|  |  | min - max | 6790 - 11989870 | 6280 - 12132090 |  |  |  |
|  |  |  |  |  |  |  |  |
| **Supplementary Table 3.3 Healthcare costs within 3-months (Patients without ONS during study period)** | | | | | |  |  |
|  |  |  |  |  |  |  |  |
|  |  |  | PS matched cohort | | | | |
|  |  |  | ONSd group | Non-ONSd group | Ratio (95%CI) | P-value | Adjusted P-value |
|  | Healthcare costs (yen) | n | 8708 | 12545 | 0.825 (0.808 - 0.842) | <0.001 | <0.001 |
|  |  | mean (SD) | 801310.5 (750819.5) | 661279.0 (634292.5) |  |  |  |
|  |  | median | 612085.0 | 505830.0 |  |  |  |
|  |  | q1 - q3 | 362900.0 - 986145.0 | 294610.0 - 826550.0 |  |  |  |
|  |  | min - max | 6790 - 11989870 | 6280 - 13909180 |  |  |  |
|  |  |  |  |  |  |  |  |
| **Supplementary Table 3.4 Healthcare costs within 6-months (Patients without ONS during study period)** | | | | | |  |  |
|  |  |  |  |  |  |  |  |
|  |  |  | PS matched cohort | | | | |
|  |  |  | ONSd group | Non-ONSd group | Ratio (95%CI) | P-value | Adjusted P-value |
|  | Healthcare costs (yen) | n | 8708 | 12545 | 0.881 (0.864 - 0.900) | <0.001 | <0.001 |
|  |  | mean (SD) | 837373.7 (778594.9) | 738109.9 (779936.8) |  |  |  |
|  |  | median | 645600.0 | 570060.0 |  |  |  |
|  |  | q1 - q3 | 386200.0 - 1019095.0 | 341950.0 - 897690.0 |  |  |  |
|  |  | min - max | 6790 - 11989870 | 7220 - 28389910 |  |  |  |
|  |  |  |  |  |  |  |  |
| **Supplementary Table 3.5 Healthcare costs within 12-months (Patients without ONS during study period)** | | | | | |  |  |
|  |  |  |  |  |  |  |  |
|  |  |  | PS matched cohort | | | | |
|  |  |  | ONSd group | Non-ONSd group | Ratio (95%CI) | P-value | Adjusted P-value |
|  | Healthcare costs (yen) | n | 8708 | 12545 | 0.961 (0.941 - 0.981) | <0.001 | <0.001 |
|  |  | mean (SD) | 882465.2 (835892.1) | 847699.5 (1056391.0) |  |  |  |
|  |  | median | 682460.0 | 654370.0 |  |  |  |
|  |  | q1 - q3 | 402910.0 - 1067000.0 | 386610.0 - 1011490.0 |  |  |  |
|  |  | min - max | 6790 - 11989870 | 7220 - 52708680 |  |  |  |

*Adjusted P values correspond to Benjamini–Hochberg–adjusted P values applied for multiple-comparison control.

Supplementary Table 4 IPTW-based sensitivity analysis of cumulative healthcare costs (in yen) over 12 months in the overall cohort and stratified by ONS prescription status during follow-up

| All cohort | Cumulative healthcare costs | 1 month | | 2 months | | 3 months | | 6 months | | 12 months | |
| --- | --- | --- | --- | --- | --- | --- | --- | --- | --- | --- | --- |
|  |  | n | mean | n | mean | n | mean | n | mean | n | mean |
|  | ONSd group | 13042 | 761831.9 | 13042 | 809136.4 | 13042 | 844560.9 | 13042 | 919261.4 | 13042 | 1012614.9 |
|  | Non-ONSd group | 513543 | 597925.7 | 513543 | 638626.9 | 513543 | 672683.8 | 513543 | 756010.8 | 513543 | 878401.5 |
|  | P-value | <0.001 |  | <0.001 |  | <0.001 |  | <0.001 |  | <0.001 |  |
|  | Adjusted P-value | <0.001 |  | <0.001 |  | <0.001 |  | <0.001 |  | <0.001 |  |
|  |  |  |  |  |  |  |  |  |  |  |  |
| Patients with ONS prescription during follow-up period | Cumulative healthcare costs | 1 month | | 2 months | | 3 months | | 6 months | | 12 months | |
|  |  | n | mean | n | mean | n | mean | n | mean | n | mean |
|  | ONSd group | 4334 | 547807.8 | 4334 | 643672.0 | 4334 | 719364.4 | 4334 | 879798.5 | 4334 | 1082959.2 |
|  | Non-ONSd group | 11569 | 557029.0 | 11569 | 637636.3 | 11569 | 710161.5 | 11569 | 891804.5 | 11569 | 1163425.3 |
|  | P-value | 0.219 |  | 0.466 |  | 0.308 |  | 0.271 |  | <0.001 |  |
|  | Adjusted P-value | 0.274 |  | 0.466 |  | 0.330 |  | 0.313 |  | <0.001 |  |
|  |  |  |  |  |  |  |  |  |  |  |  |
| Patients without ONS prescription during follow-up period | Cumulative healthcare costs | 1 month | | 2 months | | 3 months | | 6 months | | 12 months | |
|  |  | n | mean | n | mean | n | mean | n | mean | n | mean |
|  | ONSd group | 8708 | 848964.9 | 8708 | 876499.9 | 8708 | 895530.6 | 8708 | 935327.5 | 8708 | 983976.5 |
|  | Non-ONSd group | 501974 | 598884.3 | 501974 | 638650.1 | 501974 | 671805.4 | 501974 | 752828.2 | 501974 | 871721.4 |
|  | P-value | <0.001 |  | <0.001 |  | <0.001 |  | <0.001 |  | <0.001 |  |
|  | Adjusted P-value | <0.001 |  | <0.001 |  | <0.001 |  | <0.001 |  | <0.001 |  |

*Adjusted P values correspond to Benjamini–Hochberg–adjusted P values applied for multiple-comparison control.

Supplementary Table 5 Stratified analysis of cumulative healthcare costs (in yen) over 12 months according to the amount or frequency of ONS prescription during follow up period

|  |  | ONSd group | non-ONSd group | Ratio | P value | Adjusted P value |
| --- | --- | --- | --- | --- | --- | --- |
| Total amount of ONS prescription (g) during follow-up period | 0< <=1000 | 1089061.0  (193482.4) | 1474700.0  (390559.1) | 0.738 | 0.342 | 0.342 |
|  | 1000< <=7000 | 971832.2  (24976.2) | 1190331.2  (67562.3) | 0.816 | 0.001 | 0.005 |
|  | 7000< <=30000 | 944322.5  (16423.1) | 1083445.1  (52988.2) | 0.872 | 0.008 | 0.016 |
|  | 30000< | 1129722.3  (15218.0) | 1286625.9  (64102.4) | 0.878 | 0.012 | 0.016 |
| Frequency of ONS prescription during follow-up period | Not more than once in 90 days | 1051684.3  (18122.1) | 1208511.0  (46741.1) | 0.870 | 0.001 | 0.002 |
|  | More than once in 90 days | 1035309.9  (12619.4) | 1154269.4  (52415.2) | 0.897 | 0.021 | 0.021 |

*Adjusted P values correspond to Benjamini–Hochberg–adjusted P values applied for multiple-comparison control.

Supplementary Table 6 The LOS of readmission/rehospitalization, the number of outpatient physician visits and the number of emergency room visits at 360 days postdischarge

|  |  | PS matched cohort | | | | |
| --- | --- | --- | --- | --- | --- | --- |
|  |  | ONSd group | Non-ONSd group | Ratio (95%CI) | P-value | Adjusted P-value |
| LOS of readmission/rehospitalization (days) | n | 13042 | 13042 | 0.813 (0.809 - 0.817) | <0.001 | <0.001 |
|  | mean (SD) | 27.8 (55.7) | 22.6 (51.2) |  |  |  |
|  | median | 0.0 | 0.0 |  |  |  |
|  | q1 - q3 | 0.0 - 32.0 | 0.0 - 21.0 |  |  |  |
|  | min - max | 0 - 363 | 0 - 365 |  |  |  |
| Number of outpatient physician visits | n | 13042 | 13042 | 1.184 (1.170 - 1.197) | <0.001 | <0.001 |
|  | mean (SD) | 4.3 (4.4) | 5.1 (4.7) |  |  |  |
|  | median | 2.0 | 3.0 |  |  |  |
|  | q1 - q3 | 1.0 - 9.0 | 1.0 - 11.0 |  |  |  |
|  | min - max | 0 - 12 | 0 - 12 |  |  |  |
| Number of Emergency room visit | n | 13042 | 13042 | 1.077 (0.923 - 1.256) | 0.347 | 0.347 |
| / emergency call to HCP | mean (SD) | 0.0 (0.2) | 0.0 (0.2) |  |  |  |
|  | median | 0.0 | 0.0 |  |  |  |
|  | q1 - q3 | 0.0 - 0.0 | 0.0 - 0.0 |  |  |  |
|  | min - max | 0 - 1 | 0 - 1 |  |  |  |

*Adjusted P values correspond to Benjamini–Hochberg–adjusted P values applied for multiple-comparison control.

Supplementary Table 7 Healthcare costs of drugs within 6 months postdischarge

|  |  | PS matched cohort | | | |
| --- | --- | --- | --- | --- | --- |
|  |  | ONSd group | Non-ONSd group | Ratio (95%CI) | P-value |
| Healthcare costs of drugs (yen) | n | 13042 | 13042 | 0.933 (0.907 - 0.959) | <0.001 |
|  | mean (SD) | 124546.5 (257292.7) | 112010.7 (477664.1) |  |  |
|  | median | 68092.5 | 55850.3 |  |  |
|  | q1 - q3 | 32003.8 - 132367.9 | 20326.6 - 116573.8 |  |  |
|  | min - max | 652 - 6812454 | 0 - 28154071 |  |  |

Supplementary Figure 1


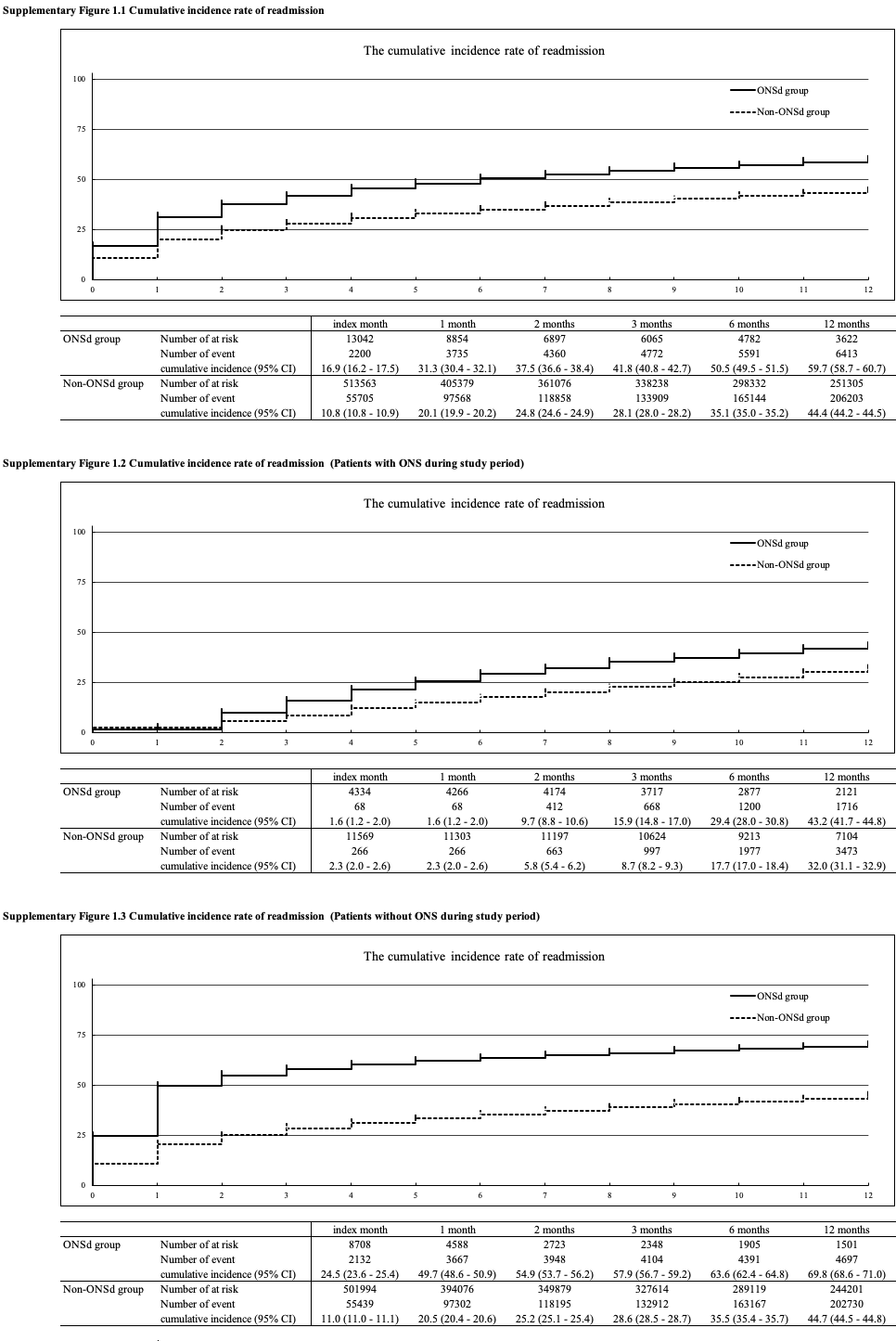


The cumulative incidence rate of readmission within 12 months after discharge, stratified by ONS prescription status.
